# Artificial intelligence – the Janus-faced tool in our hands

**DOI:** 10.1007/s11325-024-03129-7

**Published:** 2024-08-05

**Authors:** Nikolaus C. Netzer

**Affiliations:** 1 Institute of Mountain Emergency Medicine, Eurac Research, Noi Park Campus, Via Hypathia 2, 39100 Bozen, Italy; 2 Hermann Buhl Institute of Hypoxia and Sleep Medicine Research, Institute of Sport Science, University Innsbruck, Innsbruck, Austria; 3 Division of Sport Medicine, Department of Medicine, University Hospitals Ulm, Ulm, Germany

“A tool doesn’t harm people, people harm people.” The National Rifle Association (NRA) even stretches this slogan to interpret the second amendment in the Bill of Rights in a way that everybody should have the right to buy and own any kind of weapon. While this Janus-faced interpretation of using a tool is already questionable based on the original text of the second amendment, which speaks of a militia to defend the state in combination with weapon ownership, it becomes kind of absurd when it allows children in early puberty in the State of Missouri to own an AR-15 and bring it to school in their school backpack with Disney characters.

But what might be inadequate for firearms, fits our use of artificial intelligence (AI) as a Janus-faced tool, like a kitchen knife, normally extremely useful but can also be misused to injure someone, figuratively quite well. AI can help to interpret clinical symptoms faster and more extensive than a doctor’s brain and find the right diagnosis in shorter time. It does that not much differently than Dr. House does it. It scans probabilities. But out of a much larger sample base, that humans could ever do.

To give a simple example of how the Generative Pretrained Transformer (GPT) works: Finish the sentence “I put the toast in the ………..” Now ChatGPT scans a billion sentences with toast and gets a 99.99999 probability, that the toast should be put in the toaster and not in the mailbox (Generative). It memorizes this action and uses it the next time immediately without the scanning process (Pretrained). It has been pretrained that an envelope should be put in the mailbox. Now finish the sentence “I wanted to show my enemy that he is toast, so I put a toast in an envelope and put it in……” From what it had all learned, ChatGPT will finish the sentence with “………. the mailbox” (Transformer). And it can meanwhile in this form put together complicated texts, elaborate diagnoses from images etc. But it is at least in the moment generative, transforming and not fully creative to invent something completely new out of nowhere as a human like Albert Einstein did with a thought about gravity to come up with the relativity theory. And it is not powered to proof the sense of his solution. Problem is, we are not all Einsteins.

The positive aspect of AI for writing manuscripts is of course finding the best references, the best statistical methods etc. For example we ask ChatGPT a certain scientific question like “Does OSAS increase symptoms of the metabolic syndrome in geriatric patients?” If I put the key words osa, metabolic syndrome and geriatric patients into Pubmed, I get seven results. ChatGPT finds way more and I can ask it to produce a metanalysis of the results [1].

AI can help to write nursing protocols and save valuable nursing time for the care of patients in a time in which the shortage of nurses is becoming almost a desperate situation in health care in most western countries. However, already for this possible usage of AI, authors, who tested it and wrote an article about it, see possible problems of integrity and possible nonsense outcome, which if not double checked by a registered human nurse could lead to mistreatment of patients [2].

Whereas the uncontrolled use of AI here can cause real physical harm, the nonethical use of AI in composing scientific papers is more than annoying to the scientific community. The harmed here include peers, reviewers, publishers and of course the scientific interested public including media representatives, who rely on senseful non fake scientific publications [3]. “Research” papermills, businesses that publish poor or fake journal papers that seem to resemble genuine research, as well as sell authorship, put more and more fake AI composed papers out for publication and flood scientific journals, hybrid and pure open access journals alike [4]. For the peer reviewing process AI written manuscripts without the author’s disclosure of the usage of AI are a great burden. It is difficult for reviewers to detect AI composition, because the writing style is often flawless, or at least easily readable and it needs a lot of concentration, critical spirit and specific knowledge of the reviewed topic to see that the scientific conclusion of a paper is maybe nonsense or adds nothing at all to the field. On the same token reviews of papers, which are done by reviewers composed entirely by AI are annoying too. This time for authors.

I experienced a situation with a to my now best knowledge AI composed paper in two different roles. First as reviewer for a BMC journal, I experienced for the first time a mendelian randomization of a large United States database by Chinese authors not known to me, neither their institution. I was at that time not aware of maybe AI composition of papers. The conclusion sounded reasonable; the English style writing was without mistakes as seemed the statistical method. Since I found no clear reasons to reject the paper, I let it pass with minor revisions. A few months later, now back as editor in chief of Sleep and Breathing, I saw the “same” paper again, this time the author order was reversed as was the hypothesis and neither the derivation of the hypothesis nor the discussion and conclusion made scientific sense. I rejected the paper and too late I recognized my mistake to not reject the first version of the paper as reviewer of the BMC journal.

In the last few months Sleep and Breathing has received quite many papers with mendelian randomization or cross-sectional analysis of data from large United States databases, mainly NHANES. The authors were entirely from Asia. These papers were for me after my experience before under doubt of their integrity, but I had no proof for my general suspicion. Then a warning came from the Springer Publisher administration, that fake papers from research paper mills (see above) mainly with mendelian randomization of NHANES data would float around and be submitted to scientific journals. For some reviewers this warning might have come too late, and they faced the same situation as I did several month ago with the paper submitted to BMC. Therefore, there is the possibility that a few papers, which are in doubt of their scientific integrity made it through the review process at this journal.

At Sleep and Breathing we have now reacted to this actual difficult situation by not accepting papers with mendelian randomization or cross-sectional analyses from large public health databases, if these databases are not located in the corresponding authors own home country. Furthermore, we have enlarged the associated editor team by two Chinese editors, who are known to us and are recognized since years in the field of sleep medicine in order to proof the integrity of the source of the papers.

The European Commission has reacted to the problem that faces science with AI and published “Living guidelines on the responsible use of generative AI in research” [5]. The main aspects of these guidelines are keeping up the fairness in the use of AI in science and disclose any usage in grant applications and scientific publications. The guidelines do not intend to stop the progress in science, which can come with AI in a positive sense. The EU science board, which was established to develop these guidelines, had in mind to see AI as the power mentioned in the title. A tool, which can do good in many ways, but also a harmful tool if misused.

It rests the hope, that this view is adapted by all scientists and physicians worldwide.
